# Comparative analysis of different impression techniques in relation to single tooth impression

**DOI:** 10.6026/973206300161105

**Published:** 2020-12-31

**Authors:** Aman Merchant, Subhabrata Maiti, V Ashok, Dhanraj M Ganapathy

**Affiliations:** 1 Saveetha Dental College, Saveetha Institute of Medical and Technical Sciences, Saveetha University, Chennai 77, India

**Keywords:** Accuracy, Dimensional Stability, Impressions

## Abstract

It is of interest to compare the accuracy of three different impression techniques for a single tooth impression. We used 3 groups with 15 samples each in this study. Group 1: Putty and light body in a sectional stock tray; Group 2: Monophase and extra light body in a sectional stock tray; Group 3: Matrix impression technique. 15 impressions were taken of a prepared tooth on a typodont with each technique. The dimensions of the casts poured from these impression techniques were compared with the control typodont tooth. Data analysis shows that the matrix impression technique gave the best results in terms of dimensional study followed by monophase and extra light body impression technique and putty and light body impression technique gave the least accurate results. The results show that there is a statistically significant difference between the three impression techniques in terms of dimensional stability. Data analysis shows that the matrix impression technique gave the best results in terms of dimensional study followed by monophase and extra light body impression technique and putty and light body impression technique gave the least accurate results. The variations between the groups are within acceptable limits. Hence, it can be concluded that all the impression techniques will result in adequate dimensional stability and can be used in clinical scenarios.

## Background

A successful fixed dental prosthesis is dependent upon the long-term health of the periodontal tissues as well as accurately recording the finish line of the prepared tooth [1]. It is very important to record a good impression, as this record will be transferred to the lab for the fabrication of indirect restoration. A good impression technique is required to achieve an accurate restoration. Hence, a good impression forms the basis of a good prosthesis. There are different materials and techniques to record impressions. Nevertheless, whichever technique is used, the prosthesis should have a good marginal fit because an sufficient marginal fit will prompt plaque retention and leaching out of the luting cement that will cause secondary caries leading to pulpal and periodontal inflammation which will ultimately lead to failure of the prosthesis [2]. The quality of the impression is dependent on a number of factors like location of the finish line, biotype of the gingiva, sulcular bleeding etc. The advancement in materials and development in techniques is an integral part for any dental procedure. The most commonly used materials for taking an impression are vinyl polysiloxanes as they are dimensionally very stable [3], still impression technique plays a vital role in affecting the accuracy. The most commonly used method for taking impressions in the double mix technique, in which two materials with different viscosities are used [4]. In this technique, a single step or a dual step impression can be taken. Either of them can have a combination of putty and light body, putty and medium body and heavy body and light body. Some authors claim that impression materials have improved to such an extent that accuracy may be controlled more with technique than by the material itself [5]. However, other studies have indicated that the impression technique does not affect the dimensional accuracy of impressions [6]. The accuracy of the impression was much dependent on the material [7], impression tray type [8] and impression technique [9], bulk of the material [10] and other factors [11]. Hence, although these are the most commonly used techniques, it exhibits some errors, which demands for newer techniques to be introduced. One such technique is the matrix impression technique. It incorporated the attributes of the traditional techniques and at the same time overcomes the deficiencies faced with the traditional techniques like [1] registration of subgingival margins, [2] gingival retraction and relapse, [3] hemostasis and sulcular cleansing, [4] delivery of impression material subgingivally, [5] strengthening the sulcular flange of the impression, and [6] simplification for making complex impressions [12]. It works on the principle that a high viscosity material displaces the gingival tissues, which effectively flushes debris out of the sulcus. Although there are a number of studies on the accuracy of impressions related to the impression materials and impression techniques, still the controversy as to which is better remains. The types of impression techniques and the different protocols used to assess the accuracy of impressions could explain the contradictory results reported in the literature. For instance, Lee et al. [13] and Nissan et al. [14] used different quantitative analyses. Moreover, in the 1-step and 2-step techniques, only the light-body material should cover the entire preparation, but this cannot always be accomplished clinically. Therefore, it is of interest to compare the accuracy of three different impression techniques for a single tooth impression.

## Methodology

### Study design:

The present in-vitro study was conducted in The Department of Prosthodontics in Saveetha Dental College and Hospital, Chennai, India. We used 3 groups with 15 samples each in this study. Group 1: Putty and light body in a sectional stock tray; Group 2: Monophase and extra light body in a sectional stock tray; Group 3: Matrix impression technique. The overview of the study is given in Figure S1 (check with auhors) at the end of this article.

### Sample size estimation:

The sample size was estimated to be 12 in each group using G power with inputs fed from a pilot study by Nissan et al. However, the sample size was increased to 15 in each group to increase the level of significance.

### Study sample

A typodont was taken and tooth preparation was done on a mandibular first molar. The tooth was reduced by 1.5mm and a uniform 1 mm margin was prepared. The tooth was common for all the groups. After the preparation was done, points were marked on the cusps, ridges and the cervical portion to be taken as reference points to measure the dimensions of the tooth. The dimensions of the tooth were measured by six operators so as to reduce the bias. The average values were 4.78mm cervico-occlusal, 8.54mm mesiodistal and 8.14mm buccolingual. The tray is removed and the casts are poured using type IV die stone after the impression materials are set.

### Outcome Measures:

Dimensional stability was evaluated in the present study.

### Statistical analysis:

All analyses were conducted using SPSS 21 [SPSS Inc., Chicago, IL]. One way ANOVA test was performed to assess the statistical significance at 95% confidence level and 5% significance [α=.05].

## Results:

15 samples were included in each of the three groups. Group 1 has a mean value of cervico-occlusal dimension of 5.46±0.03, Group 2 has a mean value of cervico-occlusal dimension of 5.08±0.03 and Group 3 has a mean value of cervico-occlusal dimension of 4.86±0.02. Group 1 has a mean value of mesiodistal dimension of 8.76±0.02, Group 2 has a mean value of mesiodistal dimension of 8.69±0.03 and Group 3 has a mean value of mesiodistal dimension of 8.56±0.02. Group 1 has a mean value of buccolingual dimension of 8.71±0.02, Group 2 has a mean value of buccolingual dimension of 8.45±0.03 and Group 3 has a mean value of buccolingual dimension of 8.26±0.03. The results show that there is a statistically significant difference between the three impression techniques in terms of dimensional stability [p value: 0.001 [p<0.05]. When the difference was calculated between the dimensions of the control group and the experimental group, it was observed that the dimensions of Group 3 were the closest to the control group dimensions. When Tukey test was done to evaluate the association between the three experimental groups, it was observed that there was a statistically significant difference.

## Discussion:

Polyvinyl siloxane is the most popularly used impression material due to its excellent physical properties, handling characteristics, surface reproduction and dimensional stability [15]. There are several techniques to make a putty wash impression [16]. Hung et a1. [4] and Idris et a1. [17] tried various impression techniques and concluded in their studies that the accuracy of the impression is independent of the impression technique. However, some authors stated that the technique is a critical factor in influencing the accuracy of the impression [18]. In the present study, the accuracy of four impression techniques was investigated and it was found that the matrix impression technique gave the best results in terms of dimensional study followed by monophase and extra light body impression technique and putty and light body impression technique gave the least accurate results. We had compared the cervico-occlusal, mesiodistal and buccolingual dimensions of the casts poured from the three techniques and found that the dimensions from the matrix impression technique were the closest to the typodont tooth dimensions. In all the three techniques, the dimensions were found to be greater than the typodont tooth. This observation may also be explained by an expansion of stone material, although the casts were measured 24 hours after the retrieval from the impression.

Among the three techniques, the matrix impression technique was the most difficult to perform, but it gave the best results whereas, putty and light body technique was the easiest to perform, but gave the worst results. Some studies have reported that monophase impression technique gave the worst results in terms of dimensional accuracy [19] and surface defects [20] because of the high viscosity and reduced flow. In order to overcome those disadvantages, extra light body was incorporated in this technique to record the finer details, which cannot be recorded with only monophase. The matrix impression technique incorporated three basic impression techniques, but forms its basis from auto polymerizing resin technique for interim fixed restorations. It significantly improves the gingival displacement and sulcular cleansing phases. The matrix also helps in overcoming the problems faced due to gingival bleeding and contamination due to other sulcular fluids [12]. In the putty and light body technique, the light body is injected via a syringe and air is blown to allow the light body material into the gingival sulcus. In a matrix impression technique, the matrix dispenses the material into the sulcus with greater precision and consistency than a syringe but with a gentle and controlled force. This study suggests that impression technique can be a significant factor in determining the accuracy of the impression. The limitations of this study are that the sample size was less. Also, the standard typodont was used without metal abutments, which might give false readings. Hence, more number of studies is required to be conducted with proper standardization protocols to verify the results.

## Conclusion

Data analysis shows that the matrix impression technique gave the best results in terms of dimensional study followed by monophase and extra light body impression technique and putty and light body impression technique gave the least accurate results. The variations between the groups are within acceptable limits. Hence, it can be concluded that all the impression techniques will result in adequate dimensional stability and can be used in clinical scenarios.

## References

[1] Levartovsky S (2014). J Prosthodont..

[2] Hunter AJ (1990). J Prosthet Dent..

[3] Craig RG (1988). Advances in Dental Research..

[4] Hung SH (1992). J Prosthet Dent..

[5] Lee IK (1995). Oper Dent.

[6] Idris B (1995). J Prosthet Dent..

[7] Herbst D (2000). J Prosthet Dent..

[8] Brosky ME (2002). J Prosthet Dent..

[9] Vigolo P (2003). J Prosthet Dent..

[10] Lewinstein I (1993). J Oral Rehabil..

[11] Boulton JL (1996). Australian Dental Journal..

[12] Livaditis GJ (1998). J Prosthet Dent..

[13] Lee IK (1995). Oper Dent..

[14] Nissan J (2000). J Prosthet Dent..

[15] Yeh CL (1980). J Am Dent Assoc..

[16] Saunders WP (1991). J Dent..

[17] Idris B (1995). J Prosthet Dent..

[18] Morgano SM (1995). J Prosthet Dent..

[19] Caputi S (2008). The Journal of Prosthetic Dentistry..

[20] Millar BJ (1998). J Prosthet Dent..

